# SENP3-Mediated PPARγ2 DeSUMOylation in BM-MSCs Potentiates Glucocorticoid-Induced Osteoporosis by Promoting Adipogenesis and Weakening Osteogenesis

**DOI:** 10.3389/fcell.2021.693079

**Published:** 2021-06-24

**Authors:** Yongxing Zhang, Yang Chen, Hangxiang Sun, Wenkan Zhang, Lingling Zhang, Hengyuan Li, Xin Huang, Jie Yang, Zhaoming Ye

**Affiliations:** ^1^Musculoskeletal Tumor Center, Department of Orthopedics, The Second Affiliated Hospital of Zhejiang University School of Medicine, Hangzhou, China; ^2^Institute of Orthopedic Research, Zhejiang University, Hangzhou, China; ^3^Shanghai Key Laboratory for Tumor Microenvironment and Inflammation, Department of Biochemistry and Molecular Cell Biology, Shanghai Jiao Tong University School of Medicine, Shanghai, China; ^4^Department of Ultrasound, Sir Run Run Shaw Hospital, Zhejiang University School of Medicine, Hangzhou, China

**Keywords:** SENP3, SUMOylation, osteo-adipogenic differentiation, glucocorticoid-induced osteoporosis (GIOP), oxidative stress

## Abstract

Glucocorticoid-induced osteoporosis (GIOP) is the most common secondary osteoporosis and reduced bone formation was the main pathological change in GIOP. Our previous studies have shown that there was an imbalance between adipogenic and osteogenic differentiation in GIOP BM-MSCs and peroxisome proliferator-activated receptor γ2 (PPARγ2) played a vital role in this disorders. Here, we reported that there was an increase in ROS level and SENP3 expression in Dex-induced osteoporotic BM-MSCs, and enhanced adipogenesis and weakened osteogenesis in osteoporotic BM-MSCs might be caused by upregulated SENP3. Then we found that SENP3 de-SUMOylated PPARγ2 on K107 site to potentiate adipogenesis and weaken osteogenesis. These results may provide new strategy and target in the clinical diagnosis and treatment of GIOP.

## Introduction

Osteoporosis is a common metabolic disease, which refers to the systemic bone disease with reduced bone mass, disordered bone microstructure, and increased fragility, thereby increasing the risk of fracture (Schuit et al., 2004). Osteoporosis is divided into two major categories based on its cause: primary osteoporosis, which mainly includes postmenopausal osteoporosis, senile osteoporosis, juvenile osteoporosis, and secondary osteoporosis, which is mainly caused by long-term drug overuse (e.g., long-term use of glucocorticoid; Di Iorgi et al., 2008). Among them, glucocorticoid-induced osteoporosis (GIOP) is currently the most common secondary and iatrogenic osteoporosis (Whittier and Saag, 2016).

Glucocorticoids (GCs) are widely used for anti-inflammation in some immune diseases (e.g., rheumatic diseases) and immunosuppression of organ transplantation (Quax et al., 2013). However, GCs can cause a variety of side effects and complications, among which GIOP is the most important side effect and the most serious consequences of GC treatment (van Staa et al., 2002). Due to the effectiveness and specificity, the GC therapy cannot be replaced temporarily. Therefore, GIOP has become a huge medical, social, and health hotspot issue. Currently, anti-GIOP drugs include bone resorption inhibitors (e.g., bisphosphonates, selective estrogen receptor modulators), bone-forming promoters (e.g., parathyroid hormone, PTH), and other drugs (e.g., active vitamin D). However, due to off-target and other side effects, these drugs are far from satisfactory in use (Whittier and Saag, 2016; Chotiyarnwong and McCloskey, 2020). For example, gastrointestinal discomfort was often found in the patients with oral bisphosphonates, meanwhile, mandible osteonecrosis, nephrotoxicity, and the increased risk of venous thrombosis were also found in the patients with intravenous bisphosphonates (Cummings et al., 2009; Hirooka et al., 2020). Therefore, there is an urgent clinical need for a new effective treatment strategy for GIOP treatment.

Nowadays, bone marrow-derived mesenchymal stromal cells (BM-MSCs) in the development of osteoporosis has attracted more attention. Our previous studies have shown that BM-MSCs in GIOP mice had weaker osteogenic potential and a remarkable increase in adipogenic potential (Zhang Y. et al., 2015; Zhang Y. X. et al., 2015). Meanwhile, the decrease in the number and activity of osteoblasts in GIOP is accompanied by an increase in fat cells (Liu et al., 2010), and increased bone marrow adipose tissue is closely related to the oxidative stress (Chen et al., 2008).

Oxidative stress refers to the metabolic imbalance caused by reactive oxygen species (ROS) and reactive nitrogen species (RNS), among which ROS plays the major role. Compared with other differentiated cells, BM-MSCs have lower antioxidant activity and are more sensitive to oxidative stress (Orciani et al., 2010). Recent studies showed that ROS inhibits osteogenic differentiation while increases adipogenic differentiation (Higuchi et al., 2013) of BM-MSCs; exogenous H_2_O_2_ could induce human and mouse adipose precursor cells from BM-MSCs in a dose-dependent manner (Reykdal et al., 1999; Schroder et al., 2009; Higuchi et al., 2013), and ROS scavenger *N*-acetylcysteine (NAC) significantly inhibits lipogenesis in mouse MSC cells (Kanda et al., 2011). However, the mechanism of oxidative stress in BM-MSC differentiation and its role in GIOP is still unclear.

Similar to ubiquitination modification, SUMOylation is a process of catalyzing the binding of substrate to SUMO1 or SUMO2/3 by enzyme E1, E2, and E3. At the same time, SUMOylation can also be reversibly removed by SENP family. SENP-mediated de-SUMO modification can regulate protein activity, degradation, and localization (Wang and Dasso, 2009). Different from SUMO1 modification, SUMO2/3 modification was more involved in cell stress (Saitoh and Hinchey, 2000). In our previous studies, we found that the SUMO protease SENP3, which is specifically responsible for removing SUMO2/3 from substrates, is a redox-sensitive enzyme (Huang et al., 2009; Yan et al., 2010). Among the SENP family members, SENP3 is unique in its rapid response in protein level, owing to the abrogation of ubiquitin-mediated degradation after an oxidation following a mild increase in ROS (Yan et al., 2010). Previous studies showed that GC excess can cause cell stress and increase the level of ROS (Iuchi et al., 2003; Mutsaers and Tofighi, 2012). Therefore, we hypothesized that SENP3, which regulates SUMO2/3 modification under stress, may play an important role in the stress-induced imbalance of BM-MSC differentiation in GIOP.

Recent studies have shown that SENP3-meditated deSUMOylation played an important role in bone remodeling. SENP3 promoted osteogenic differentiation by de-SUMOylating RbBP5 in human dental follicle stem cells (DFSCs) (Nayak et al., 2014), and our study also showed that SENP3 could inhibit osteoclastogenesis by deSUMOylating IRF8, which was the negative regulator of NFATc1 (Zhang et al., 2020). Here, we demonstrated that there were increased SENP3 expression, enhanced adipogenic, and weakened osteogenic potential in BM-MSCs of GIOP mice. *Senp3* knockdown in BM-MSCs rescued osteogenic differentiation by weakening adipogenesis. Our study implied that SENP3-mediated deSUMOylation in a ROS-dependent manner participated in the balance between osteogenic and adipogenic differentiation in BM-MSCs, and SENP3 might be regarded as a therapeutic target in GIOP in the future.

## Materials and Methods

### Animal and Ethics Statement

C57BL/6 WT mice were purchased from the Shanghai SLAC Laboratory Animal Co. Ltd. 8- to 12-week-old mice were used for experiments. All mice were maintained under specific pathogen-free (SPF) condition in a barrier-sustained facility and provided with sterile food and water. C57BL/6 SENP3^+/–^ mice were generated as described previously (Lao et al., 2018). Animal experiments were carried out in strict accordance with the regulations in the Guide for the Care and Use of Laboratory Animals issued by the Ministry of Science and Technology of the People’s Republic of China. The protocol was approved by the Institutional Animal Care and Use Committee of the Second Affiliated Hospital of Zhejiang University School of Medicine (Permit Number: AIRB-2021-143). All surgery was performed under sodium pentobarbital anesthesia, and every effort was made to minimize suffering.

### Glucocorticoid-Induced Osteoporosis Mouse Model

The male mice were divided into different groups and were dosed once daily with subcutaneous injection with dexamethasone (Dex) phosphate (1 mg/kg; Sigma, St. Louis, MO, United States) for 4 weeks as described (Shi et al., 2015); meanwhile, subcutaneous injection with saline as control. For NAC injection, mice received 400 mg/kg of NAC (*N*-acetylcysteine) in phosphate-buffered saline (PBS). Administration of NAC was performed as a slow intravenous bolus (60 s) through a tail vein catheter. Throughout the dosing period, the mice were weighed to examine the effect of dosing on body weight. The animals were then sacrificed with an intraperitoneally injected overdose of sodium pentobarbitone (100 mg/kg). The femur was removed by dissection for micro-computerized tomography (microCT) analysis. Mouse BM-MSCs were harvested for further experiments. The treatment protocol was approved by the Ethics Committee and the Animal Research Committee, the Second Affiliated Hospital of Zhejiang University School of Medicine (Permit Number: AIRB-2021-143).

### Skeletal Phenotyping

All imaging was performed in a blinded fashion as described (Farr et al., 2017). Quantitative analysis of distal femoral metaphysis were performed by Hangzhou Yue Bo and Shanghai Saixin Biological Technology Co., Ltd. Using two-dimensional (2D) data from scanned slices, 3D analysis was used to calculate morphometric parameters at distal femoral metaphysis (100 slices) defining trabecular and cortical bone microarchitecture, including trabecular bone volume fraction (BV/TV;%), trabecular number (Tb.N; 1/mm), trabecular thickness (Tb.Th; mm), and cortical thickness (Ct.Th; mm). All μCT parameters were derived using the manufacturer’s protocols.

### Bone Marrow-Derived Mesenchymal Stromal Cell Isolation and Culture

Enzymatic digestion of bone marrow cells and CFU-F cultures were performed as described previously (Suire et al., 2012; Zhang et al., 2020). Briefly, intact marrow plugs were flushed from the long bones and subjected to two rounds of enzymatic digestion at 37°C for 15 min each. The digestion buffer contained 3 mg/ml of type I collagenase (Worthington), 4 mg/ml of dispase (Roche Diagnostic), and 1 U/ml of DNase I (Sigma) in HBSS with calcium and magnesium. The cells were resuspended in staining medium (HBSS + 2%FBS) with 2 mM EDTA to stop the digestion. Freshly dissociated single-cell suspensions were plated in 10-cm plates (5 × 10^6^ cells/dish) with α-MEM, 20% FBS, 10 mM ROCK inhibitor (Y-27632, TOCRIS), and 1% penicillin/streptomycin. The culture medium was changed on the second day after plating to wash out macrophages, then changed every 3–4 days.

### Adipogenic Differentiation and Oil Red O Staining

Adipogenic differentiation induction was performed as described previously (Watanabe et al., 2015). Briefly, when MSCs reached confluence, they were fed with complete adipogenic hormone cocktail: DMEM supplemented with 10% FBS, 10 μg/ml of insulin (I5500, Sigma), 0.5 mM 3-isobutyl-1-methylxanthine (IBMX, I5879, Sigma) and 1 μM Dex (D4902, Sigma). The start point of differentiation was referred as day 0. Cells were then incubated in the same medium containing 5 μg/ml of insulin. After 2 days, the medium was replaced with a conditioned medium. Subsequently, the conditioned medium was changed regularly every 2 days. Intracellular lipid accumulation was evaluated using Oil Red O staining.

### Osteogenic Differentiation, Alkaline Phosphatase Staining, and Alizarin Red Staining

Osteogenic differentiation induction was performed as described previously (Watanabe et al., 2015). Briefly, when cells reached confluence, they were fed with complete osteogenic differentiation medium: α-MEM supplemented with 10% FBS, 50 μg/ml of L-ascorbic acid (A5960, Sigma), and 4 mmol/L of sodium glycerophosphate (G9422, Sigma). The start point of differentiation was referred as day 0. The medium was replaced every other day. Then, after 7 days intracellular alkaline phosphatase (ALP) activity and after 21 days intracellular calcium nodules were evaluated using ALP staining and Alizarin Red staining, respectively.

### siRNA, Plasmid, Mutagenesis, and Transfection

The siRNA specific for SENP3 and non-specific control siRNA oligonucleotides were synthesized and used as previously described (Han et al., 2010). The plasmids of Flag-SENP3 and Flag-SENP3 mutant C532A were constructed and used previously (Yan et al., 2010). The plasmid Flag-PPARγ2 was kindly provided by Prof. Zhaoyuan Hou (Shanghai Key Laboratory of Tumor Microenvironment and Inflammation, Shanghai Jiao Tong University School of Medicine, China). Based on Flag-PPARγ2, Flag-PPARγ2 Lys/Arg mutant (K107R) was generated by Shanghai TranSheepBio Biological Technology Co., Ltd.

Attractene Transfection Reagent (ATR, QIAGEN, Germany) was used for transfection reagent as described (Lao et al., 2018). Briefly, the day before transfection, an appropriate amount of cells were seeded into the plate with the density 60–80% during transfection. The siRNA–ATR/plasmid–ATR mixture was added to the plate, gently shaken, and mixed; the plate was placed in a 37°C incubator overnight. Culture medium was changed after 12 h and then incubated for another 24–48 h.

### Quantitative RT-PCR

This method was as previously described (Zhou et al., 2016). Total RNA was isolated from cells and tissues using Trizol reagent (Invitrogen), and cDNA was synthesized using Takara reagent. Quantitative real-time PCR was conducted using SYBR Green (Roche, Switzerland) on the LightCycler 480 system. GAPDH expression was used as internal control. The primer sequences used were as follows:

**TABLE 1:** 

GAPDH	Forward	5′-TACAGCAACAGGGTGGTGGAC-3′
	Reverse	5′-TGGGATAGGGCCTCTCTTGCT-3′
PPARγ2	Forward	5′-TCGCTGATGCACTGCCTATG-3′
	Reverse	5′-GAGAGGTCCACAGAGCTGATT-3′
C/EBPα	Forward	5′-GCCGAGATAAAGCCAAACAAC-3′
	Reverse	5′-GACCCGAAACCATCCTCTG-3′
Adiponectin	Forward	5′-CCGCTTATGTGTATCGCTCAG-3′
	Reverse	5′-CGGAATGTTGCAGTAGAACTTG-3′
FABP4	Forward	5′-GCGTGGAATTCGATGAAATCA-3′
	Reverse	5′-CCCGCCATCTAGGGTTATGA-3′
Runx2	Forward	5′-GACTGTGGTTACCGTCATGGC-3′
	Reverse	5′-ACTTGGTTTTTCATAACAGCGGA-3′
Ostrix	Forward	5′-ATGGCTCGTGGTACAAGGC-3′
	Reverse	5′-GCAAAGTCAGATGGGTAAGTAGG-3′
OCN	Forward	5′-CGGCCCTGAGTCTGACAAA-3′
	Reverse	5′-GCCGGAGTCTGTTCACTACCTT-3′
		

### Immunoblotting

Immunoblotting (IB) was performed using the routine methods as described before (Huang et al., 2009). The antibodies against SUMO2/3 (4971), SENP3 (5591) were purchased from Cell Signaling Technology (Beverly, MA, United States). The antibody against RH (34610) was purchased from Qiagen. The antibodies against Flag (F3165) and β-actin (A5441) were purchased from Sigma-Aldrich (Saint Louis, MO, United States). The antibody against PPARγ2 (sc-7273) was purchased from Santa Cruz Biotech Inc.

### Flag Immunoprecipitation Assay

The method was as previously described (Zhou et al., 2016). Briefly, transfected cells were lysed in a lysis buffer (50 mM Tris-HCl, pH 7.4, 150 mM NaCl, 1 mM EDTA and 1% Triton X-100). Anti-FLAG M2 Affinity Gel (A2220, Sigma) was added to the cell lysates and incubated overnight at 4°C. The beads were washed four times in the lysis buffer. After the last wash, Flag-tagged proteins were eluted in elution buffer [lysis buffer containing cocktail protease inhibitor (Roche) and 20 mM NEM (Sigma)] and then subjected to IB.

### Co-immunoprecipitation Assay

The method was performed as previously described (Zhou et al., 2016). Cells were lysed and sonicated in RIPA buffer (Thermo Scientific, Pittsburgh, PA, United States) at 4°C for 30 min, then centrifuged at 13,000 × *g* at 4°C for another 30 min. The cell lysates were pre-cleared by adding 40 μl of Protein-A/G agarose beads (IP05, Calbiochem, Temecula, CA, United States) per 1 ml and incubating at 4°C for 30 min. The protein-A/G beads were then removed by centrifugation. Specific antibodies were mixed with the supernatants overnight at 4°C. Protein-A/G agarose beads were added to the lysates, and the mixture was incubated under shaking for 4 h at 4°C. NEM at 20 mM was included in the IP buffer to ensure SUMOylation to be conserved during manipulation. The beads were washed three times, mixed with loading buffer and examined by SDS–PAGE and IB analyses.

### Reactive Oxygen Species Detection

2′,7′-Dichlorofluorescin diacetate (DCFH-DA, Sigma-Aldrich, 35848) was used as an ROS capturing reagent as previously described (Zhou et al., 2016; Li et al., 2021). Briefly, cells were incubated with 5 μM DCFH-DA in serum-free culture media at 37°C for 1 h, and washed with PBS before flow cytometry.

### Statistics Analysis

Statistical significance was calculated by Student’s *t*-test for two-sample comparisons, one-way ANOVA was used for multiple comparisons in software SPSS 19.0, and Tukey’s test was used to find significant differences in ANOVA. Statistical significance was analyzed on data from at least three independent experiments. *p*-Values < 0.05 were defined as significant. All data are presented as mean ± SEM unless otherwise specified.

## Results

### Enhanced Adipogenesis and Weakened Osteogenesis Were Found in Bone Marrow-Derived Mesenchymal Stromal Cells of Glucocorticoid-Induced Osteoporosis Mouse Model

**FIGURE 1:**
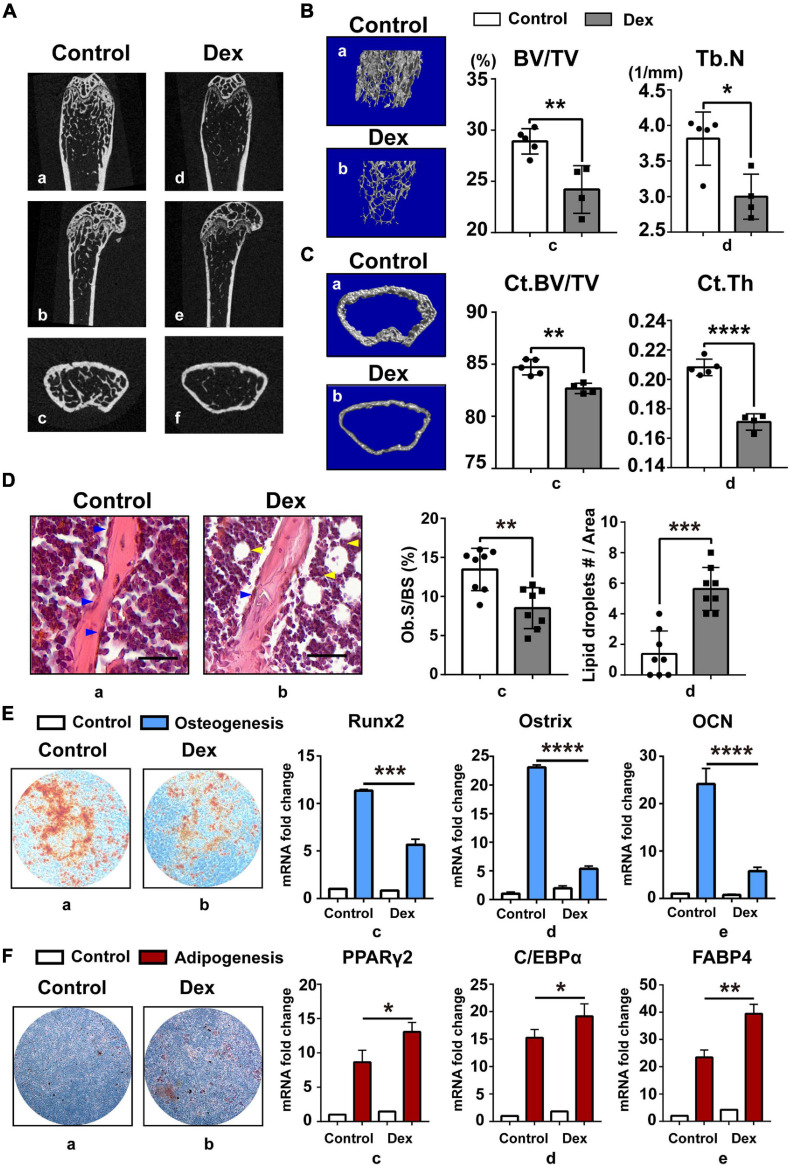
Enhanced adipogenesis and weakened osteogenesis were found in BM-MSCs of GIOP mouse model. **(A)** Represented images of the coronal **(a,d)**, sagittal **(b,e)**, and transverse **(c,f)** of distal femur in 8–12-week-old mice, as measured by micro-CT 2D restoration (*n* = 5, control group; and *n* = 4, Dex injection group)**; (B)** represented images of three-dimensional (3D) cancellous restoration **(a,b)**, bone volume/tissue volume ratios (%) (BV/TV) **(c)**, trabecular number (Tb.N) **(d)** of the distal femur cancellous bone in 8–12 week-old mice, as measured by micro-CT scan (*n* = 5, control group; and *n* = 4, Dex injection group). **(C)** Represented images of three-dimensional (3D) cortical restoration **(a,b)**, cortical bone volume/tissue volume ratios (%) (Ct.BV/TV) **(c)**, cortical thickness (Ct.Th) **(d)** of the distal femur cortical bone in 8–12 week-old mice, as measured by micro-CT scan (*n* = 5, control group; and *n* = 4, Dex injection group). **(D)** HE staining of distal femur on paraffin-embedded bone sections in control group **(a)** and Dex injection group **(b)** mice (scale bar, 100 mm; blue arrow heads point to osteoblasts, yellow arrow heads point to lipid droplets); osteoblast surface/bone surface ratios (Ob.S/BS) **(c)** and the number of lipid droplets (lipid droplets #/Area) **(d)** are shown on the right. **(E)** Arizarin Red staining of BM-MSCs in control **(a)** and Dex injection group **(b)** after 21-day osteogenic induction; Runx2 **(c)**, Ostrix **(d)**, OCN **(e)** mRNA levels in day 0 and day 7 after osteogenesis, as measured by qPCR. **(F)** Oil Red O staining of BM-MSCs in control **(a)** and Dex injection group **(b)** after 14-day adipogenic induction; PPARγ2 **(c)**, C/EBPα **(d)**, FABP4 **(e)** mRNA levels in day 0 and day 7 after adipogenesis, as measured by qPCR. Data are shown as the mean ± SEM. *p < 0.05, **p < 0.01, ***p < 0.005, ****p < 0.001. All the data were obtained from at least three independent experiments.

In the current study, GIOP mouse model was established by subcutaneously injecting Dex for 4 weeks as described (Shi et al., 2015). Saline-injected mice were used as control and defined as “normal mouse.” Based on skeletal phenotype analysis, we found that long-term injection of GC can cause severe bone loss (Figure 1A) in mice and GIOP mice have less trabecular bone (Figure 1B) and thinner cortical bone (Figure 1C). Then we found that there were less osteoblasts and more lipid droplets (Figure 1D) in GIOP mice after H&E staining. Next, BM-MSCs were isolated from bone marrow in normal and GIOP mice as described above, and they were induced to undergo *in vitro* osteogenic (Figure 1E) and adipogenic (Figure 1F) differentiation. After Alizarin Red and Oil Red O staining, less calcium nodules and more intracellular lipid droplets were observed in BM-MSCs of GIOP mice compared with normal BM-MSCs. At days 0 and 7, total RNA was harvested to detect adipogenic marker genes including PPARγ2, C/EBPα, and FABP4, and osteogenic marker genes including Runx2, Ostrix, and OCN by q-PCR. Without osteogenic or adipogenic induction, there was no difference in mRNA abundance of these markers between normal and osteoporotic BM-MSCs. However, upon induction, the remarkable downregulation of Runx2, Ostrix, and OCN, and upregulation of PPARγ2, C/EBPα, and FABP4 were observed in osteoporotic BM-MSCs compared with normal ones (Figures 1E,F), supporting the data from the mouse model skeletal phenotype analysis. These data suggested that osteoporotic BM-MSCs had enhanced adipogenic and weakened osteogenic potential than normal BM-MSCs.

### Dex Damaged Osteogenesis While Compensating for Enhanced Adipogenesis *in vitro* by Upregulating Cellular Reactive Oxygen Species Level

**FIGURE 2:**
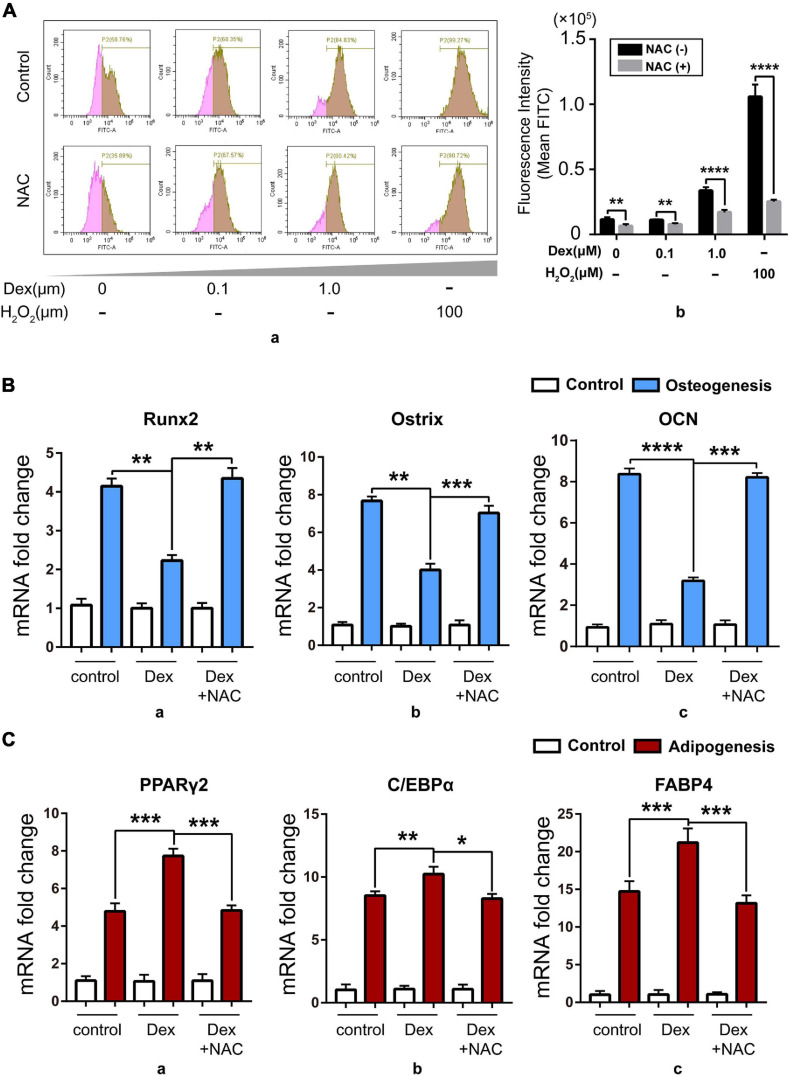
Dex damaged osteogenesis while compensating for enhanced adipogenesis *in vitro* by upregulating cellular ROS level. **(A)** The cellular ROS level in BM-MSCs with 0, 0.1, and 1.0 μM Dex treatment for 24 h detected by flow cytometry after DCFH-DA staining (100 μM, 15 min) with/without 5 mM NAC treatment, and 100 μM H_2_O_2_ treatment for 30 min was used as positive control. Data were shown in peak map **(a)** and histogram **(b)**; **(B)** The expression of osteogenic marker genes including Runx2 **(a)**, Ostrix **(b)**, OCN **(c)** in BM-MSCs of control group and Dex-treated group with/without NAC after 7-day osteogenic induction was detected by Q-PCR; **(C)** The expression of adipogenic marker genes including PPARγ2 **(a)**, C/EBPα **(b)**, fatty acid binding protein 4 (FABP4, **c**) in BM-MSCs of control group and Dex-treated group with/without NAC after 7-day adipogenic induction was detected by Q-PCR; Data are shown as the mean ± SEM. *p < 0.05, **p < 0.01, ***p < 0.005, ****p < 0.001. All the data were obtained from at least three independent experiments.

In order to explore the relation between excess GC and the imbalance of osteo-adipogenesis of BM-MSCs, we studied the intracellular oxidative stress level during BM-MSC differentiation. Iuchi et al. (2003) found that GC can increase the level of cellular ROS. Based on that report, we isolated BM-MSCs from 8- to 12-week-old C57BL/6 mice and then treated BM-MSCs with a range of concentration of Dex from 0, 0.1, and 1 μM for 24 h with 100 μM H_2_O_2_ as positive control, and found that cellular ROS level rose after Dex treatment nearly in a dose-dependent manner and can both be reversed by NAC, a ROS scavenger (Figure 2A). Next, we treated isolated BM-MSCs with Dex (1 μM) and Dex (1 μM) + NAC (5 mM) during osteogenic and adipogenic differentiation, with solvent DMSO as control. Data about the expression of osteogenic and adipogenic marker genes showed that Dex could damage the osteogenic potential (Figure 2B) while compensating for enhanced adipogenic potential (Figure 2C). Meanwhile, the ROS scavenger NAC could rescue the phenotype, which meant that the cellular ROS increased by Dex might be the trigger of the imbalance of osteo-adipogenesis of BM-MSCs. Our previous studies have shown that cellular SENP3 can accumulate under mild oxidative stress (Yan et al., 2010), thus we speculated that SENP3 in BM-MSCs increased by exogenous GC may have an impact on BM-MSC differentiation and GIOP progress.

### Upregulated Cellular SENP3 Under Oxidative Stress in Bone Marrow-Derived Mesenchymal Stromal Cells Can Promote Adipogenesis and Inhibited Osteogenesis

**FIGURE 3:**
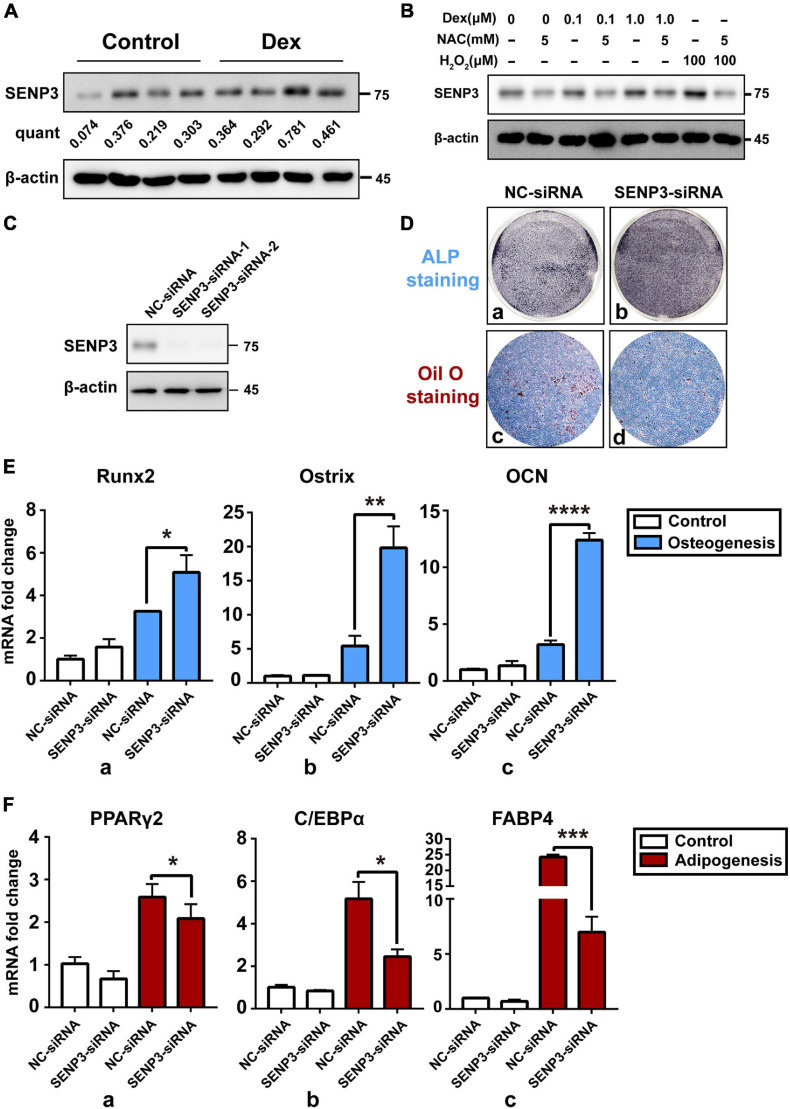
Upregulated cellular SENP3 under oxidative stress in BM-MSCs can promote adipogenesis and inhibited osteogenesis. **(A)** The protein level of SENP3 in BM-MSCs of control and GIOP mice were shown by Western Blotting and the ratio of SENP3/β-actin was listed below; **(B)** the protein level of SENP3 in BM-MSCs after 0, 0.1, 1.0 μM Dex treatment for 24 h with/without 5 mM NAC treatment, 100 μM H_2_O_2_ treatment for 30 min was used as positive control; **(C)** the expression of SENP3 in BM-MSCs transfected by NC-siRNA and SENP3-siRNA for 36 h was shown by Western Blot; **(D)** The ALP staining and Oil Red O staining of BM-MSCs transfected by NC-siRNA **(a,c)** and SENP3-siRNA **(b,d)** after osteogenic induction for 7 days and adipogenic induction for 14 days; **(E)** the expression of osteogenic marker genes including Runx2 **(a)**, Ostrix **(b)**, OCN **(c)** in BM-MSCs with NC-siRNA and SENP3-siRNA transfected in day 0 and day 7 after osteogenic induction was detected by Q-PCR; **(F)** The expression of adipogenic marker genes including PPARγ2 **(a)**, C/EBPα **(b)**, fatty acid binding protein 4 (FABP4, **c**) in BM-MSCs with NC-siRNA and SENP3-siRNA transfected in day 0 and day 7 after adipogenic induction was detected by Q-PCR. Data are shown as the mean ± SEM. *p < 0.05, **p < 0.01, ***p < 0.005, ****p < 0.001. All the data were obtained from at least three independent experiments.

In order to identify the relation between SENP3 and BM-MSC differentiation and GIOP progress, SENP3 in BM-MSCs from normal and GIOP mice was evaluated by western blot. Data showed that SENP3 was upregulated in osteoporotic BM-MSCs (Figure 3A). Thus we treated BM-MSCs with Dex in a range of concentration from 0, 0.1, and 1.0 μM and found that the protein of cellular SENP3 can be increased in a dose-dependent manner (Figure 3B). Meanwhile, NAC could also inhibited the upregulation of SENP3 by Dex. Meanwhile, we also analyzed the mRNA and protein levels of SENP3 in BM-MSCs during adipogenesis and osteogenesis, and we found that the protein levels of SENP3 increased and there was a higher gain during BM-MSC adipogenesis (Supplementary Figures 1A,C), while the mRNA levels of SENP3 in BM-MSCs remained nearly unchanged at day 7 after osteogenesis and adipogenesis (Supplementary Figures 1B,D). Then in order to explore the correlation between SENP3 and osteogenic/adipogenic differentiation, BM-MSCs transfected by NC-siRNA and SENP3-siRNA (Figure 3C) were conducted with ALP staining after osteogenic induction for 7 days and Oil Red O staining after adipogenic induction for 14 days, and we found that there was deeper ALP staining in SENP3–siRNA transfected BM-MSCs, and the lipid droplets in NC–siRNA transfected BM-MSCs were much more and bigger than those in SENP3–siRNA transfected ones (Figure 3D). We also evaluated the expression of osteogenic and adipogenic marker genes and found that ALP staining and Oil Red O staining was also consistent with the results of the q-PCR, which showed that during BM-MSC differentiation, osteogenic marker genes (e.g., Runx2, Ostrix, and OCN) were upregulated (Figure 3E), while adipogenic marker genes (e.g., PPARγ2, C/EBPα, and FABP4) were downregulated (Figure 3F) after SENP3 knockdown. It is well known that SENP family has an impact on the biological process by deSUMOylation. Since our previous study (Zhang Y. et al., 2015) has shown that peroxisome proliferator-activated receptor γ2 (PPARγ2) plays an important role in the balance of adipogenesis and osteogenesis in BM-MSCs. Meanwhile, Ohshima et al. (2004) has reported SUMO-1 modification of PPARγ in 2004. Based on that, we decided to detect the SUMO2/3 modification of PPARγ2 in BM-MSCs during osteogenic and adipogenic differentiation.

### PPARγ2 Can Be SUMO2/3 Modified During Adipogenic Induction and deSUMOylated by SENP3

**FIGURE 4:**
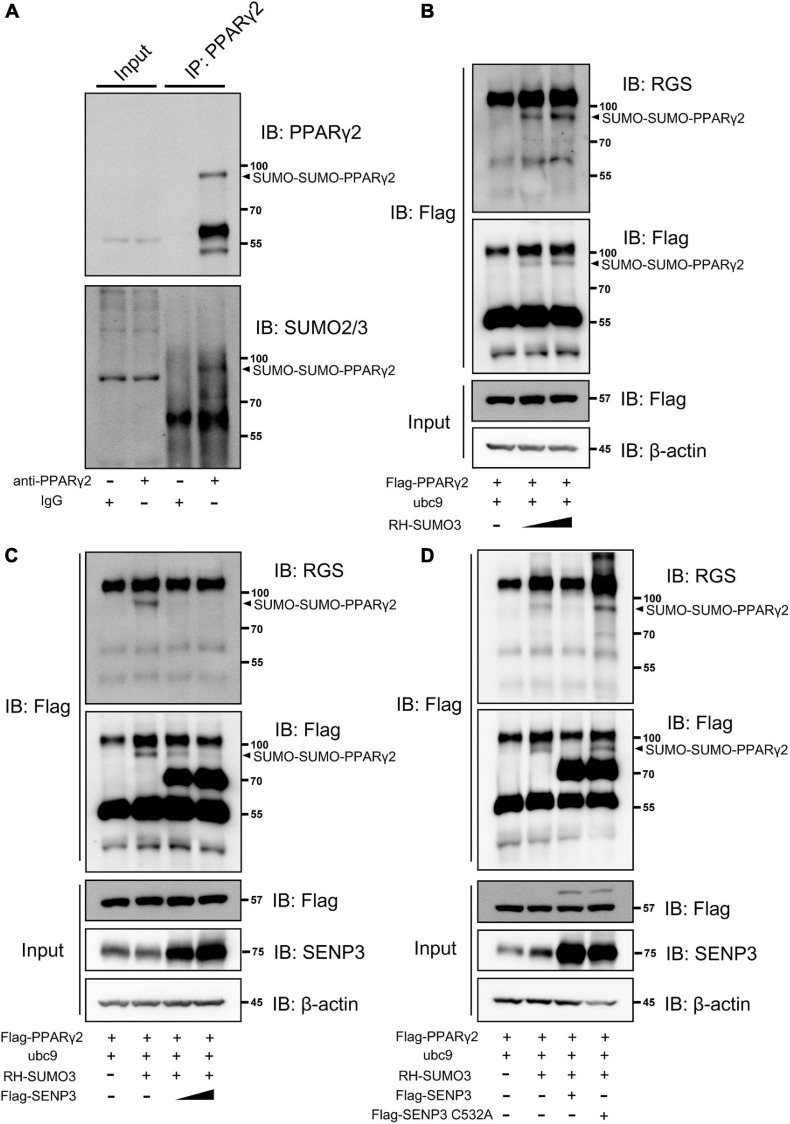
Peroxisome proliferator-activated receptor γ2 (PPARγ2) can be SUMO2/3 modification during adipogenic induction and deSUMOylated by SENP3. **(A)** The endogenous SUMO2/3 modification of PPARγ2 in BM-MSCs during adipogenesis. Whole-cell lysates were prepared from BM-MSCs undergone adipogenic differentiation at day 3 and were incubated with antibody specific to PPARγ2. The co-eluted proteins were detected by using PPARγ2 and SUMO2/3 antibody. **(B)** PPARγ2 can be modified by SUMO3 when co-expressed in HEK293T cells in dose-dependent manner; Flag-PPARγ2, ubc9 and RH-SUMO3 were co-expressed in HEK293T cells and co-IP assays were performed with Flag and RGS antibody; **(C)** the SUMOylation of PPARγ2 can be removed by SENP3; Flag-PPARγ2, ubc9, Flag-SENP3, and RH-SUMO3 were co-expressed in HEK293T cells and co-IP assays were performed with Flag and RGS antibody; **(D)** SENP3 mediated de-SUMOylation depended on its active site cysteine 532 (C532); Flag-PPARγ2, ubc9, Flag-SENP3 (WT and C532A mutant) and RH-SUMO3 were co-expressed in HEK293T cells and co-IP assays were performed with Flag and RGS antibody.

We detected the SUMO2/3 modification of PPARγ2 in BM-MSCs during adipogenic induction and found that there was a modification band of SUMO2/3 modification (Figure 4A). We next tested whether PPARγ2 could be deSUMOylated by SENP3 in HEK293T cells. SUMOylation of PPARγ2 was examined by using Flag-bead IP in SUMO3 overexpression cells. SUMO3 conjugation of PPARγ2 displayed a prominent band on the gel at a 97-kDa molecular weight (MW) in a dose-dependent manner, thus, indicating that two SUMO conjugate was bound to PPARγ2 (approximately 57 kDa in MW) (Figure 4B). Wild-type SENP3 (WT) was able to remove SUMO3 from PPARγ2 in a dose-dependent manner (Figure 4C), whereas the SENP3 mutant (C532A, lacking enzymatic activity) was not able to do so (Figure 4D). Therefore, we confirmed that the SUMO2/3 modification of PPARγ2 existed during BM-MSC adipogenesis, and this progress depended on SENP3.

### SENP^ +/−^ Mice Were Less Likely to Suffer Severe Glucocorticoid-Induced Osteoporosis Probably With Undisturbed Osteogenesis by Dex-Induced Reactive Oxygen Species Upregulation

**FIGURE 5:**
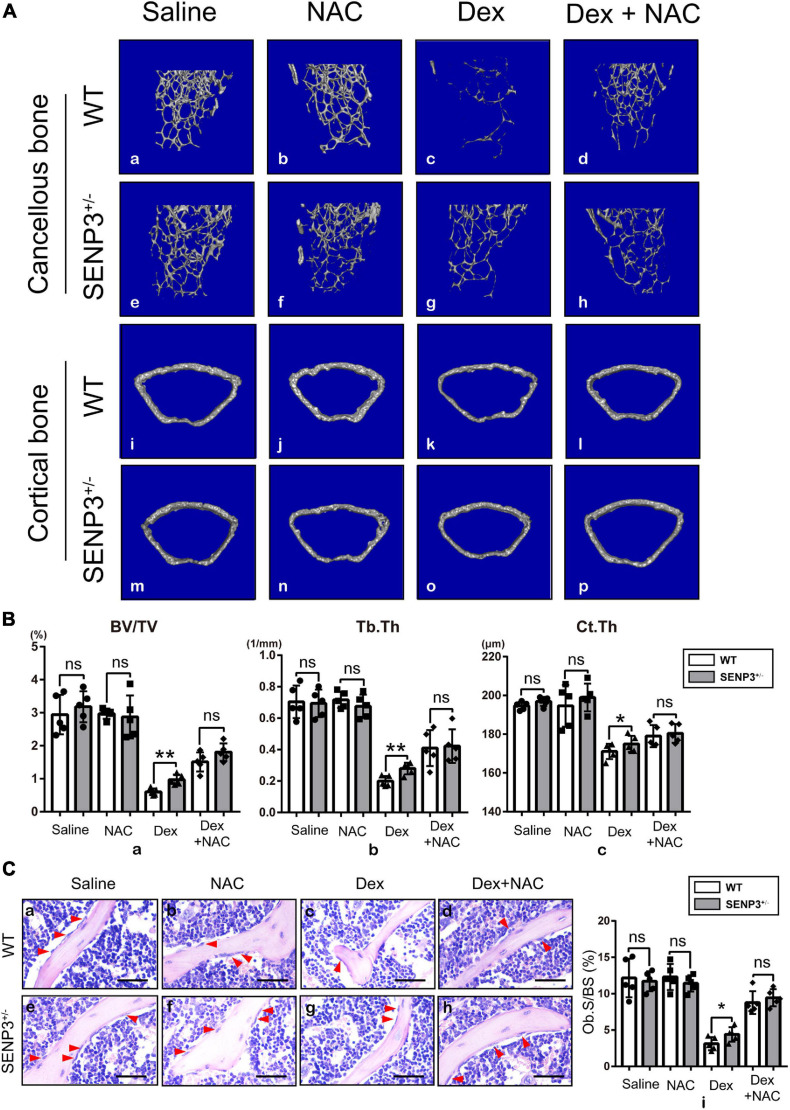
SENP^ +/−^ mice were less likely to suffer severe GIOP probably with undisturbed osteogenesis by Dex-induced ROS upregulation. **(A)** Represented images of three-dimensional (3D) cancellous bone restoration **(a–h)** of the distal femur cancellous bone in 8–12 week-old WT and SENP3^ +/−^ mice with saline (*n* = 5, **a,e**), NAC alone (*n* = 5, **b,f**), Dex alone (*n* = 5, **c,g**) and Dex + NAC (*n* = 5, **d,h**), and three-dimensional (3D) cortical bone restoration **(i–p)** of the distal femur cortical bone in 8–12-week-old WT and SENP3^ +/−^ mice with saline (*n* = 5, **a,e**), NAC alone (*n* = 5, **b,f**), Dex alone (*n* = 5, **c,g**) and Dex + NAC (*n* = 5, **d,h**); **(B)** Trabecular bone volume/tissue volume ratios (%) (BV/TV, **a**), trabecular thickness (Tb.Th, **b**) and cortical thickness (Ct.Th, **c**) the distal femur cancellous and cortical bone in 8–12-week-old WT and SENP3^ +/−^ mice with saline (*n* = 5), NAC alone (*n* = 5), Dex alone (*n* = 5), and Dex + NAC (*n* = 5). **(C)** HE staining **(a–h)** of distal femur on paraffin-embedded bone sections in in 8–12-week-old WT and SENP3^ +/−^ mice with saline, NAC alone, Dex alone and Dex + NAC (scale bar, 100 mm; red arrow heads point to osteoblasts); osteoblast surface/bone surface ratios (Ob.S/BS) **(i)** is shown on the right. Data are shown as the mean ± SEM. *p < 0.05, **p < 0.01, ns: not significant. All the data were obtained from at least three independent experiments.

Since SENP3^–/–^ mice were embryonally lethal, the study was performed using SENP3 knockout heterozygotes (SENP3^ +/−^) mice. We found that long-term use of GC can cause severe osteoporosis in mice but SENP3^ +/−^ mice were less likely to suffer severe bone loss after GC induction; however, NAC injection might rescue the GIOP progress with eliminating Dex-induced ROS upregulation (Figure 5A). Based on skeletal phenotype analysis, after GC injection, and compared with WT mice, SENP3^ +/−^ mice have a higher trabecular bone volume fraction (BV/TV;%), trabecular thickness (Tb.Th, μm) and cortical thickness (Ct.Th, μm); meanwhile, NAC injection could rescue the Dex-induced bone loss as the ROS scavenger (Figure 5B). Then H&E staining and histological analysis showed that there were more osteoblasts on the surface of trabecular bone in SENP3^ +/−^ mice compared with WT ones; at the same time, NAC also could rescue the phenotype of Dex-induced osteogenic deficiency (Figure 5C).

### The SUMOylation Reduced the Activity of PPARγ2 Through K107 Site and Weakened Adipogenic Differentiation

**FIGURE 6:**
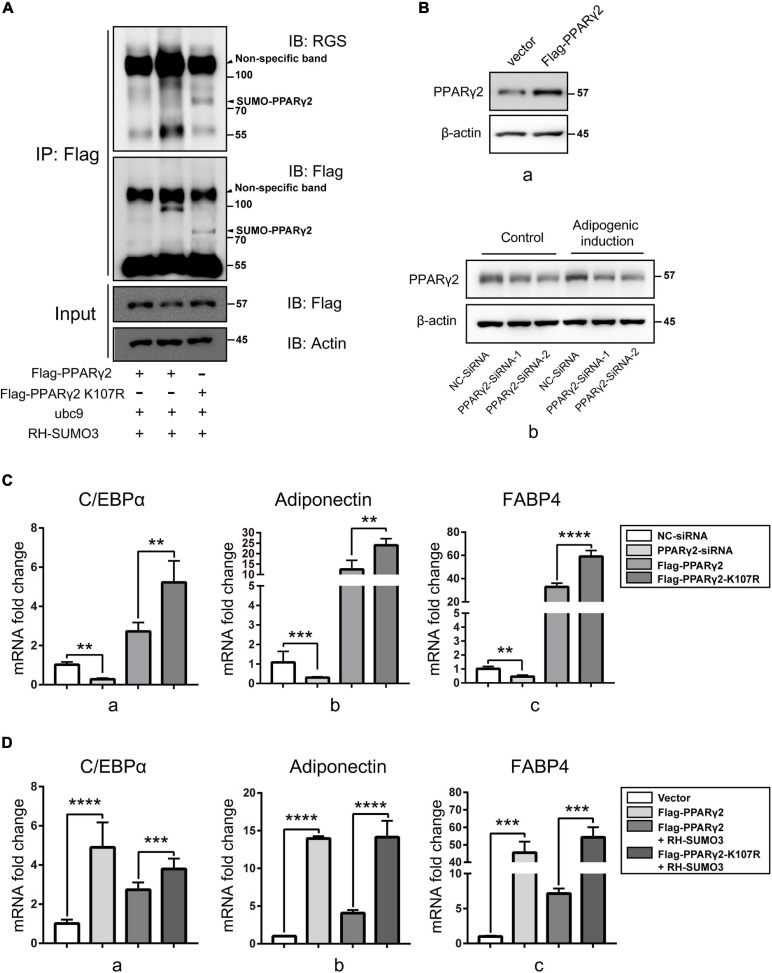
The SUMOylation reduced the activity of PPARγ2 through K107 site and weakened adipogenic differentiation. **(A)** The SUMOylation of PPARγ2 was relied on Lys residues of 107 and 395. Flag-PPARγ2, Flag-PPARγ2-K107R, ubc9 and RH-SUMO3 were co-expressed in HEK293T cells and co-IP assays were performed with Flag and RGS antibody. **(B)** The expression of PPARγ2 in ST2 cells transfected by Flag-PPARγ2 for 24h **(a)** and NC-siRNA and PPARγ2-siRNA for 36 h **(b)** were detected by Western Blot. **(C)** NC-SiRNA, PPARγ2–SiRNA, Flag-PPARγ2, and Flag-PPARγ2-K107R were transfected in mouse mesenchymal stromal cell line ST2 cells and the expression of adipogenic marker genes C/EBPα **(a)**, adiponectin **(b)**, and FABP4 **(c)** was analyzed by Q-PCR after 7-day adipogenic induction. **(D)** Flag-PPARγ2, Flag-PPARγ2-K107R, and RH-SUMO3 were transfected in ST2 cells and the expression of adipogenic marker genes C/EBPα **(a)**, adiponectin **(b)**, and FABP4 **(c)** was analyzed by Q-PCR after 7-day adipogenic induction. Data are shown as the mean ± SEM. **p < 0.01, ***p < 0.001; ****p < 0.0001. All the data were obtained from at least three independent experiments.

Reports have shown that Lys residues of 107 and 395 were the SUMOylation sites of PPARγ2 (Geiss-Friedlander and Melchior, 2007), while only K107 SUMOylation is related to the activity of PPARγ2 in adipogenic activation (Pascual et al., 2005). To verify the effect of SUMOylation of PPARγ2 on its activity, we constructed plasmids for expression of mutants in which Lys residues of 107 were replaced by Arg, thus, preventing SUMOylation. SUMOylation of PPARγ2 was again examined using Flag IP. The results clearly showed that PPARγ2 was conjugated with SUMO3 at K107 and K395 because the WT PPARγ2 was pulled down with SUMO3 conjugates, displaying the SUMO band, and the K107R mutant lacked this band with another band about 77 kDa forming, which was probably the SUMO3 modification at K395 (Figure 6A). These data verified PPARγ2 as the substrate of SENP3 and K107/395 as the site of PPARγ2 SUMO3 modification. Then we transfected NC-siRNA, PPARγ2-siRNA, WT PPARγ2 (Flag-PPARγ2), and the K107R mutant (Flag-PPARγ2-K107R) in mouse mesenchymal stromal cell line ST2 cells and then conducted adipogenic differentiation induction for 7 days (Figure 6B). Q-PCR results showed that the expression of adipogenic genes was upregulated in WT PPARγ2 and the K107R mutant overexpression group, while we found that the activity of the K107R mutant to promote the expression of adipogenic genes was much higher than that of WT PPARγ2 (Figure 6C). At the same time, we transfected WT PPARγ2 (Flag-PPARγ2) and the K107R mutant (Flag-PPARγ2-K107R) with/without RH-SUMO3 during 7-day adipogenic induction in ST2 cells. Then we found that RH-SUMO3 overexpression has little effect on adipogenesis in the K107R mutant overexpression group, while it could inhibit adipogenesis in the WT PPARγ2 overexpression group (Figure 6D). Thus, we speculated that SENP3 might promote adipogenesis during oxidative stress by deSUMOylating PPARγ2 in BM-MSCs.

## Discussion

**FIGURE 7:**
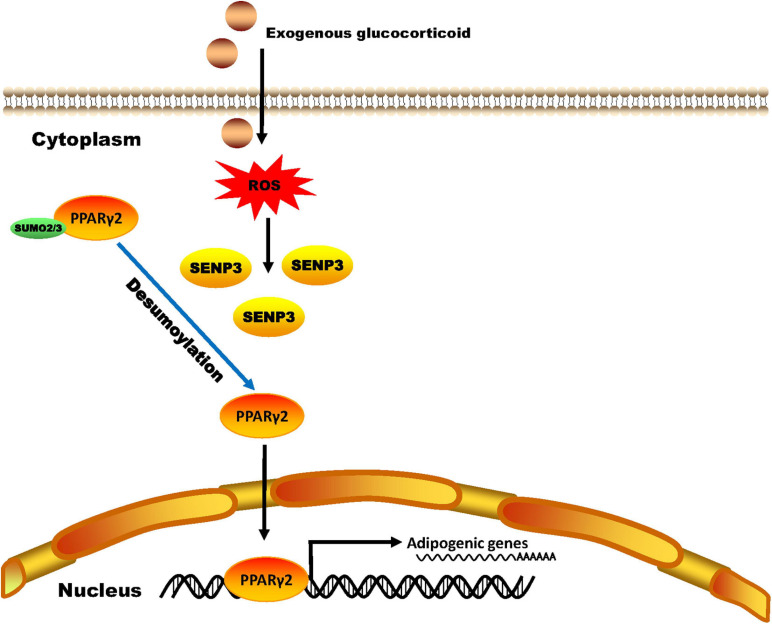
A schematic model of SENP3 function in glucocorticoid-induced PPARγ2 signaling. Exogenous glucocorticoid increases the cellular ROS level of BM-MSCs and the protein level of SENP3. SENP3 deSUMOylates SUMO2/3 modification of PPARγ2 on Lys residue 107 site and enhanced its activity to promote the expression of adipogenic marker genes such as C/EBPα, adiponectin and FABP4. Therefore, SENP3 potentiates adipogenesis in BM-MSCs and aggravate glucocorticoid-induced osteoporosis under oxidative stress.

In the present study, we reported a previously undescribed role of the SUMO protease SENP3 in the imbalance between osteogenesis and adipogenesis of BM-MSCs in the GIOP, and also reported first SUMO2/3 modification of PPARγ2 under oxidative stress (Figure 7).

Glucocorticoid-induced osteoporosis is the most common secondary osteoporosis (Whittier and Saag, 2016). Research showed that reduced bone formation was the main pathological change in GIOP (Weinstein, 2011). GC can upregulate the expression of DKK-1 and sclerostin, and then inhibit Wnt signaling in osteoblasts and cause a reduction of bone formation (Yao et al., 2008; Hayashi et al., 2009). Apart from that, our previous study has shown that there was an imbalance between adipogenic and osteogenic differentiation in GIOP BM-MSCs and PPARγ2 played a vital role in this disorders (Zhao et al., 2013; Zhang Y. et al., 2015; Zhang Y. X. et al., 2015). PPARγ is a member of the PPAR family of transcriptional factors and nuclear receptors and plays a pivotal part in cell fate determination, lipid biosynthesis, inflammation, and insulin sensitivity (Rosen and MacDougald, 2006). There are two main PPARγ variants, PPARγ1 and PPARγ2. PPARγ1 is expressed in a wide range of tissues, including the liver, skeletal muscle, adipose tissue and bone, while PPARγ2 contains 30 additional amino acids in its N-terminus compared with PPARγ1, is expressed mainly in adipogenic progenitor cells and more important in adipogenic differentiation (Kawai et al., 2010). Since Ohshima et al. (2004) has reported PPARγ could be SUMO1 modified in 2004, many researches have been conducted to explore the effect of SUMOylation on the activity of PPARγ (Floyd and Stephens, 2004; Shimizu et al., 2006; Dutchak et al., 2012). Compared with SUMO1, which is mostly conjugated firmly to substrates, SUMO2/3 modification is more likely to be regulated by cell stress (Saitoh and Hinchey, 2000; Herrmann et al., 2007). As the main protease of de-SUMO2/3, our research group has reported several substrates of SENP3 during oxidative stress (Wang et al., 2012). Since oxidative stress has been reported in reduced bone formation and bone loss (Almeida and O’Brien, 2013), we intended to explore the correlation between SENP3 and development of GIOP.

Here, we found that SENP3 potentiates the adipogenesis in BM-MSCs and GIOP. Although it is reported that GC in high dose can directly activate the expression of adipogenic differentiation genes like PPARγ2 (Ou et al., 2014), while we still found that Dex-induced ROS could weaken osteogenic potential and enhance adipogenic potential, and ROS scavenger NAC could rescue this phenotype (Figure 2). Meanwhile, we found that SENP3 expression was upregulated in GIOP BM-MSCs and SENP3 knockdown might increase osteogenic potential and inhibited adipogenic potential in BM-MSCs (Figure 3). Then we also found that the protein levels of SENP3 increased and there was a higher gain during BM-MSC adipogenesis (Supplementary Figures 1A,C), while the mRNA levels of SENP3 in BM-MSCs remained nearly unchanged at day 7 after osteogenesis and adipogenesis (Supplementary Figure 1), which means that there might be ROS upregulation during osteogenesis and adipogenesis, which induced intracellular SENP3 accumulation depending on transcriptional regulation, while adipogenic-related regulatory factors were more sensitive and involved in oxidative stress-induced SENP3 and SUMOylation regulation. Apart from that, the SUMO2/3 modification of PPARγ2 in BM-MSCs during adipogenic differentiation was found (Figure 4), which means there might be a post-translation modification (PTM) on PPARγ2 when treated with GC, and its activity was changed under oxidative stress. In other words, there may be another regulation on the activity of PPARγ2 during adipogenic differentiation apart from GC-induced transcription activation as we found previously (Zhang Y. et al., 2015). Not beyond our expectations, we found that in the GIOP model, NAC could alleviate the GIOP progress, and SENP3^ +/−^ mice were less likely to suffer severe bone loss (Figure 5). Finally, we found that SENP3 might promote adipogenesis during oxidative stress by deSUMOylating PPARγ2 at the K107 site in BM-MSCs (Figure 6), and PPARγ2 K107R mutant could produce the phenotype of SENP3-induced imbalance between osteogenesis and adipogenesis under oxidative stress.

According to our previous study, upregulated adipogenesis can be coupled with reduced osteogenic differentiation. Not surprisingly, during this present research, we found that there was also a decrease in the density of calcium nodule after Alizarin Red staining and consistent change in the expression of osteogenic-related genes (Figures 1E,F), and this phenomenon was also rescued in SENP3 knockdown experiment (Figure 3D). However, this phenomenon might be inconsistent with Muller’s study (Nayak et al., 2014), which showed that the SENP3-DLX3 pathway regulating osteogenic differentiation of human stem cells and SENP3 could promote osteogenic differentiation in human dental follicle stem cells (hDFSCs) through epigenetic control. For this phenomenon, we guess that due to the complexity of osteogenic differentiation, which contains crossing multiple pathways, the regulation of osteogenesis might be somehow different in BM-MSCs and DFSCs, which could lead to the difference in osteogenic potential when responding to oxidative stress.

SUMOylation of transcriptional regulators mostly correlates with the inhibition of transcription (Geiss-Friedlander and Melchior, 2007). Two functional SUMOylation sites have been identify for PPARγ2, lysine 107 in the AF1 region and lysine 395 in the AF2 region. Reports showed that conjugation of SUMO to lysine K395 is not involved in the regulation of PPARγ-targeting genes, but in the trans-repression of inflammatory genes by PPARγ2 in macrophages (Pascual et al., 2005). Thus, here, we merely constructed the Lys 107 mutant and found that SUMOylation in K107 repressed the activity of PPARγ2 in adipogenic differentiation, which was consistent with our previously studies (Huang et al., 2009; Han et al., 2010; Zhou et al., 2016) showing that SUMOylation usually repressed the activity of modified proteins.

In summary, we used *Senp3* knockdown and SENP3^ +/−^ mice model to explore the role of SENP3 in BM-MSCs in GIOP, and to find out whether SENP3 participates in GIOP progression by regulating BM-MSC differentiation, and confirming that PPARγ2 can be the de-SUMO2/3 substrate of SENP3 and its role in the balance between osteogenic and adipogenic differentiation in BM-MSCs during oxidative stress. However, there were some limitations in this study. For example, we need SENP3 specific knockout mice in BM-MSCs to identify the role of SENP3 in BM-MSCs in GIOP, which are being progressed now. Still, our present study explores the role of oxidative stress in musculoskeletal metabolic diseases and post-translational modification of specific protein in stem cell differentiation, providing a prospect of the new strategy for GIOP treatment.

## Data Availability Statement

The original contributions presented in the study are included in the article/Supplementary Material, further inquiries can be directed to the corresponding author/s.

## Ethics Statement

The animal study was reviewed and approved by Institutional Animal Care & Use Committee of the Second Affiliated Hospital of Zhejiang University School of Medicine.

## Author Contributions

YZ, YC, and ZY designed and performed the experiments and wrote the manuscript. HS, WZ, LZ, HL, XH, and JY assisted with the experiments. YZ and HS analyzed the skeletal phenotyping and tissue histology. YZ, JY, and ZY designed and arranged figures. JY and ZY checked the English grammar and polished the English language in the manuscript. All the authors contributed to the article and approved the submitted version.

## Conflict of Interest

The authors declare that the research was conducted in the absence of any commercial or financial relationships that could be construed as a potential conflict of interest.

## Supplementary Material

Supplementary Figure 1 The protein levels of SENP3 in BM-MSCs was upregulated while the mRNA levels of SENP3 remained nearly unchanged during adipogenesis and osteogenesis. The protein levels of SENP3 in BM-MSCs of control group and after 7-day adipogenic **(A)** and osteogenic **(C)** induction were shown by Western Blotting and the ratio of SENP3/β-actin was listed below; the mRNA levels of SENP3 in BM-MSCs of control group and after 7-day adipogenic **(B)** and osteogenic **(D)** were analyzed by Q-PCR. Data are shown as the mean ± SEM, ns: not significant. All the data were obtained from at least three independent experiments.
