# Supplementary figures and images for: SENP3-Mediated PPARγ2 DeSUMOylation in BM-MSCs Potentiates Glucocorticoid-Induced Osteoporosis by Promoting Adipogenesis and Weakening Osteogenesis
Source: Front Cell Dev Biol. 2021 Jun 24;9:693079. doi: 10.3389/fcell.2021.693079 (PMC8266396; doi:10.3389/fcell.2021.693079)

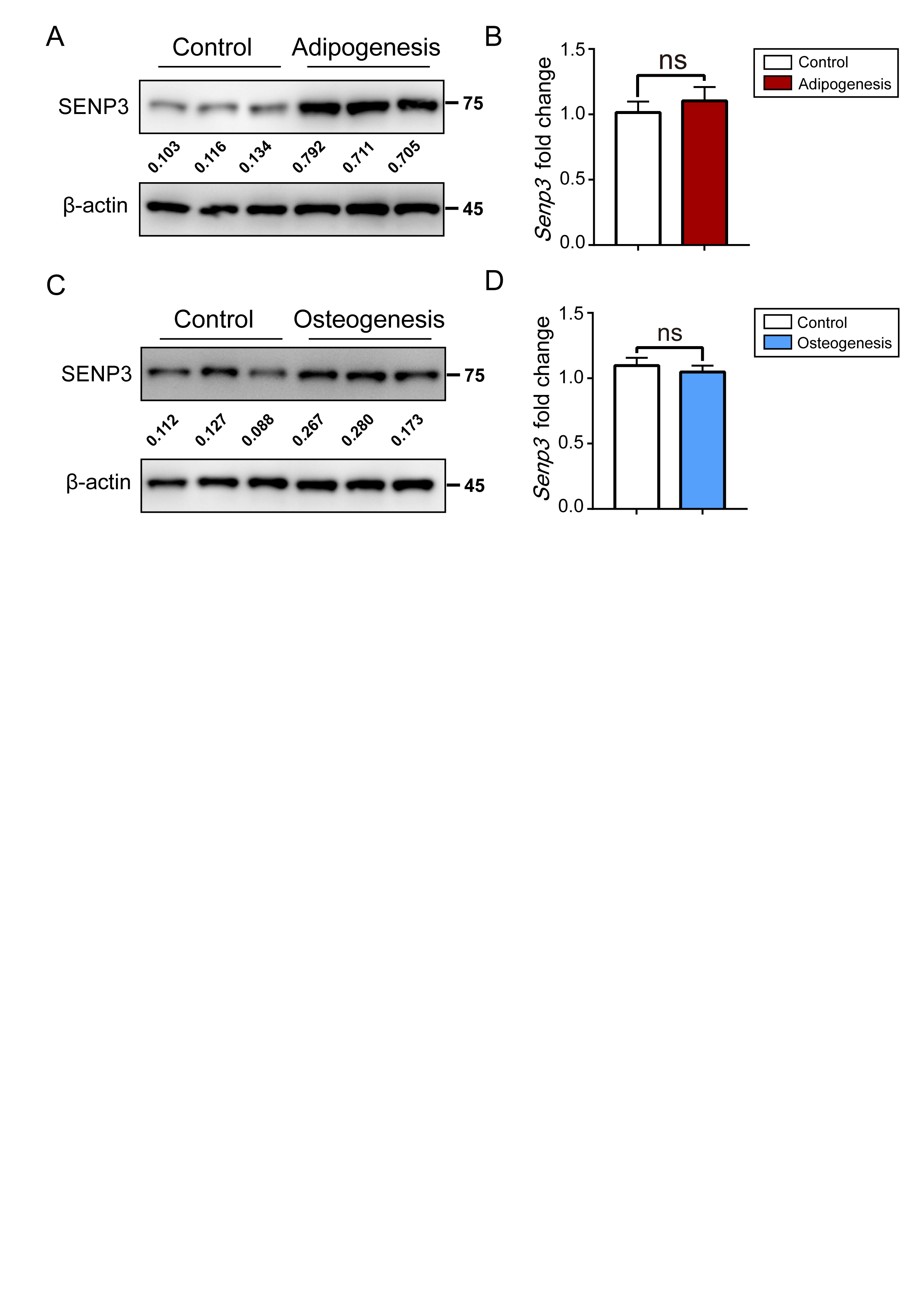

Supplement: Supplementary Figure 1 — The protein levels of SENP3 in BM-MSCs was upregulated while the mRNA levels of SENP3 remained nearly unchanged during adipogenesis and osteogenesis. The protein levels of SENP3 in BM-MSCs of control group and after 7-day adipogenic (A) and osteogenic (C) induction were shown by Western Blotting and the ratio of SENP3/β-actin was listed below; the mRNA levels of SENP3 in BM-MSCs of control group and after 7-day adipogenic (B) and osteogenic (D) were analyzed by Q-PCR. Data are shown as the mean ± SEM, ns: not significant. All the data were obtained from at least three independent experiments. [file Image_1.tif]
